# Plaque vulnerability of coronary artery lesions is related to left ventricular dilatation as determined by optical coherence tomography and cardiac magnetic resonance imaging in patients with type 2 diabetes

**DOI:** 10.1186/1475-2840-12-102

**Published:** 2013-07-11

**Authors:** Mathias Burgmaier, Michael Frick, Ana Liberman, Simone Battermann, Martin Hellmich, Walter Lehmacher, Agnes Jaskolka, Nikolaus Marx, Sebastian Reith

**Affiliations:** 1 Department of Internal Medicine I, University Hospital of the RWTH Aachen, Aachen, Germany; 2 Institute of Medical Statistics, Informatics and Epidemiology, University of Cologne, Cologne, Germany; 3 Interdisciplinary Center of Clinical Research (IZKF), University Hospital of the RWTH Aachen, Aachen, Germany

**Keywords:** Type 2 diabetes mellitus, Cardiac magnetic resonance imaging, Optical coherence tomography, Minimal fibrous cap thickness, Coronary plaque morphology

## Abstract

**Background:**

Patients with type 2 diabetes are at increased risk for both, left ventricular (LV)-dilatation and myocardial infarction (MI) following the rupture of a vulnerable plaque. This study investigated the to date incompletely understood relationship between plaque vulnerability and LV-dilatation using optical coherence tomography (OCT) and cardiac magnetic resonance imaging (CMR) in patients with type 2 diabetes and stable coronary artery disease.

**Methods:**

CMR was performed in 58 patients with type 2 diabetes, in which 81 coronary lesions were investigated using OCT.

**Results:**

A decreased minimal fibrous cap thickness (FCT) of coronary lesions was associated with an increase of several CMR-derived parameters including LV-end diastolic volume (LVEDV, r = 0.521, p < 0.001), LV-end diastolic diameter (r = 0.502, p < 0.001) and LV-end systolic volume (r = 0.467, p = 0.001). Similar results were obtained for mean FCT.

Furthermore, patients with dilated versus non-dilated LV differed significantly in several cardiovascular risk factors including previous MI (47.1% vs. 14.6%, p = 0.009), HDL-cholesterol (40.35 ± 5.57 mg/dl vs. 45.20 ± 10.79 mg/dl, p = 0.029) and smoking (82.4% vs. 51.2%, p = 0.027). However, minimal FCT is associated to LV-dilatation independent of previous MIs (odds ratio 0.679, p = 0.022).

Receiver-operating curve analysis demonstrated that CMR-derived LVEDV predicts plaque vulnerability with low-moderate diagnostic efficiency (area under the curve 0.699) and considerate specificity (83.3%) at the optimal cut-off value (159.0 ml).

**Conclusion:**

These data suggest that vulnerability of coronary lesions is associated with LV-dilatation in high risk patients with type 2 diabetes. CMR may be a useful adjunct to the risk-stratification in this population. Future studies are warranted to investigate potential mechanisms linking plaque vulnerability and LV-dilatation.

## Background

Patients with type 2 diabetes mellitus are at increased risk for both, left ventricular (LV)-dilatation as well as the presence of vulnerable coronary plaques [1-6]. Specifically, lesions from patients with type 2 diabetes are recognized to have a lower minimal thickness of the fibrous cap which overlies a lesion’s necrotic lipid core [4-6]. Thus, coronary plaques of diabetic patients are more prone to plaque rupture with subsequent myocardial infarctions (MI) [7].

Diabetes mellitus has also been recognized to be an independent risk factor for heart failure and left ventricular dilatation [3,8]. However, the relationship between plaque vulnerability and LV-dilatation is incompletely understood and may be particularly important in high risk patients with type 2 diabetes.

Optical coherence tomography (OCT) and intravascular ultrasound (IVUS) are the only in vivo imaging techniques available to determine plaque composition including the determination of the fibrous cap thickness (FCT) of coronary artery lesions [9]. OCT is a novel intravascular imaging modality with a 10-fold higher resolution than IVUS which uses the reflection of light and allows visualization and quantification of intraluminal dimensions as well as microstructures of the atheromatous plaque and the quantification of the FCT [10]. As such, we have recently used OCT to investigate associations between plaque morphology and hemodynamic relevance [11] as well as the incidence of stent edge dissections in coronary lesions [12]. However, as both IVUS and OCT are invasive techniques and harbor patient risks [13-15], their positive value in a clinical situation must be balanced against potential adverse effects.

Cardiac magnetic resonance imaging (CMR) is a non-invasive imaging technique which allows the exact determination of cardiac dimensions. Whereas echocardiography is currently the most widely used imaging technique for the assessment of LV-dilatation, CMR may be superior for the assessment of several parameters including LV-ejection fraction (LVEF), LV-end-diastolic diameter (LVEDD), LV-end-diastolic volume (LVEDV) and LV-end-systolic volume (LVESV) [16,17] particularly in frequently overweight and obese patients with type 2 diabetes.

In this study, we sought to investigate the relationship between OCT-derived plaque morphology including the FCT and CMR-derived LV-dimensions in cardiovascular high risk patients with type 2 diabetes.

## Methods

### Study population

A total of 81 de novo coronary lesions were investigated in 58 patients with stable coronary artery disease and type 2 diabetes mellitus planed for elective coronary angiography at the Department of Internal Medicine I, University Hospital of the RWTH Aachen, Germany. The indication for coronary angiography was based either on CMR-imaging suggestive for ischemia and/or typical symptoms of stable coronary artery disease. Patients were recruited into this study between August 2011 and June 2013. Quantitative coronary angiography, OCT and CMR imaging, laboratory testings and clinical history taking were performed in all patients.

Inclusion criteria were stable angina pectoris with an at least 40% coronary stenosis, known type 2 diabetes, age > 30 years and written informed consent to the study protocol. Exclusion criteria were left main coronary artery stenosis, graft stenosis, acute coronary syndromes (defined as the absence of elevated creatine kinase, persistent angina at the time of coronary intervention and electrocardiographic changes suggestive for ischemia at rest), hemodynamic or rhythmic instability, acute or chronic renal insufficiency (serum creatinine level > 1.5 mmol/l), systemic acute or chronic infections, pregnancy, anti-inflammatory medications such as steroids and chronic total occluded, severely tortuous or calcified vessels, which did not allow the safe advancement of the OCT catheter.

The study was approved by the local Ethics Committee and is in accordance with the Declaration of Helsinki on ethical principles for medical research involving human subjects.

### CMR image acquisition and analysis

CMR imaging was performed using a 1.5 Tesla magnetic resonance scanner (Achieva, Philips Healthcare, Best, The Netherlands), equipped with a 5-element cardiac synergy coil for signal reception and a vector-electro-cardiogram for cardiac synchronization. Image acquisition was performed during short repetitive end-expiratory breathholding. LV-function was assessed using balanced turbo field echo cine imaging with retrospective gating (repetition time 3.3 ms, echo time 1.6 ms, flip angle 60°; 35 phases per cardiac cycle; spatial resolution: 1.5 × 1.5 × 8.0 mm^3^) in standard cardiac geometries (3 standard long axis geometries (4-chamber, 2 chamber, left ventricular outflow tract) and in 3 short axis geometries (apical, mid-cavity, basal)).

Image-analysis was performed using a dedicated CMR-workstation (ExtendedWorkspace, Philips Healthcare, Best, The Netherlands). LVEDV, LVESV, LV-stroke volume and LVEF were determined from the long axis 4 chamber view using the “area length ejection fraction”- method. LVEDD, septal and lateral wall thickness were derived from the end-diastolic basal short axis geometry. LVEDV was normalized to body surface area (BSA) and the one-sided 95% tolerance interval of previously published CMR data was used as a cut-off for LV-dilatation (males 87.04 ml/m^2^; females 77.40 ml/m^2^) [18,19]. CMR-imaging was performed up to 24 hours before OCT image acquisition.

### OCT image acquisition and analysis

For OCT image acquisition of the target vessel we used the Frequency Domain-OCT C7XR system and the DragonFly catheter (St. Jude Medical, LightLab Imaging Inc, Westford, Massachusetts, USA) as previously described [11,12]. Target lesion identification was based on coronary angiogram with an at least 40% diameter stenosis on QCA suitable for coronary intervention and confirmed by additional information from CMR stress testing.

**Figure 1:**
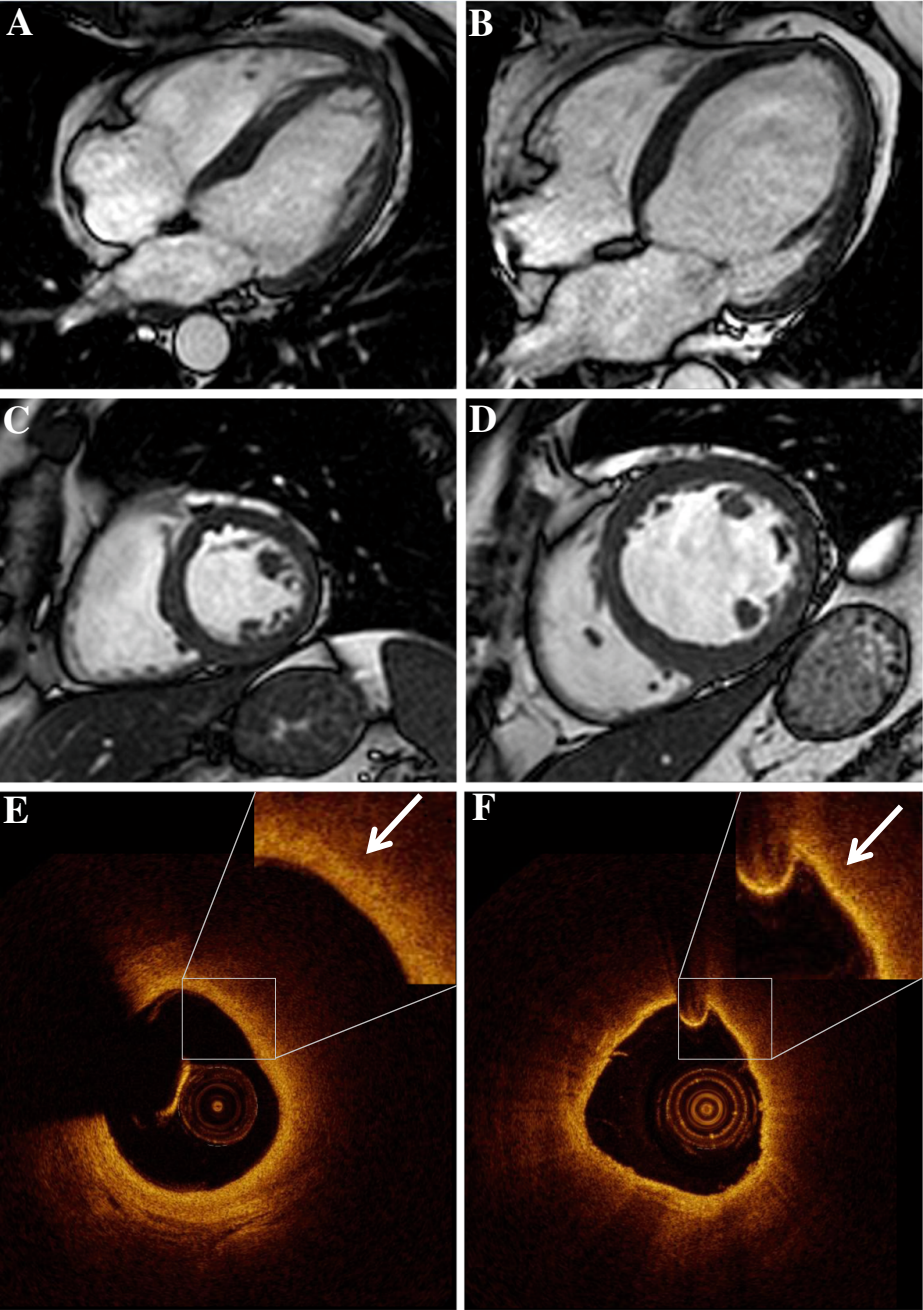
**CMR and the respective OCT images of patients with non-dilated (A, C, E) and dilated (B, D, F) left ventricle.** Displayed are representative pictures of the long axis four-chamber view **(A, B)**, the short axis **(C, D)** and the corresponding OCT-images of lipid-rich plaques **(E, F)** showing the fibrous cap in high power view (arrow).

The OCT-image analysis was performed by 2 independent observers throughout the entire lesion frame by frame in a 0.2 mm interval using the proprietary software provided by LightLab Imaging and in adaptation to the recently published consensus for qualitative and quantitative assessment [13]. Intraluminal OCT-derived parameters were assessed as previously described [11,12]. Images were analysed using validated criteria for OCT plaque characterisation and the quantification of the FCT [20,21]. In 15 patients the quantification of the FCT was not possible due to the absence of a lipid-rich plaque. A fibrous cap was defined as a signal-rich homogenous region overlying a lipid core, characterized by a diffusely bordered signal-poor region (Figure 1). FCT was measured three times at the thinnest point of each plaque and the average was calculated. Subsequently, the FCT has been quantified in a 0.2 mm interval throughout the length of the lipid plaque. The minimal FCT was defined as the minimal distance between the arterial lumen and the inner border of the lipid pool, the mean FCT as the average over the entire length of the lipid plaque. The minimal FCT to define plaque vulnerability was adjusted to 80 μm based on currently published data [22]. In lipid-rich plaques (lipid plaque >90° in any of the cross-sectional images within the plaque) the lipid arc was measured at every 1 mm interval throughout the entire length of the lipid plaque and the values were averaged. Lipid plaque length was measured in the longitudinal OCT view. The presence of plaque rupture, thrombus, calcification and fibrous plaques was noted.

### Statistical analysis

All statistical analysis was performed with SPSS Statistics (IBM Corp., Armonk, NY, USA). Categorical variables are expressed as n and percentage. Continuous variables are expressed as mean ± standard deviation. The data were analysed on a per-patient basis for clinical characteristics and on a per-stenosis basis for lesion morphology and the statistical test did not account for the correlation of multiples plaques within patients. Continuous variables were compared with *t*-test. Pearsons χ-squared test was used to compare nominal variables. Linear and non-linear regression analysis was performed to determine the association between CMR-derived LVEDV, LVESV and LVEDD and both minimal and mean FCT on a per stenosis basis. Uni- and multivariable logistic regression analysis was performed to calculate the odds ratio (OR) for minimal and mean FCT to predict LV-dilatation. Sensitivity, specificity, positive predictive value, negative predictive value and optimal cut-off values were calculated from the receiver-operating characteristic (ROC) curve to predict FCT ≤ 80 μm. Values with the highest Youden-index (sensitivity + specificity - 1) were identified as optimal cut-off-values. A classification of the diagnostic efficiency of OCT parameters according to the values of the area under the curve (AUC) was used as described elsewhere [23]. A p-value < 0.05 was regarded as statistically significant.

## Results

### Patient characteristics

We examined 58 patients with 81 lesions of at least 40% diameter stenosis as determined by quantitative coronary angiography in this study. There were no peri- or postprocedural complications associated with the use of either OCT or CMR. For patient characteristics, CMR and OCT data please refer to Tables 1 and 2.

**Table 1:** Patient characteristics

	
**Clinical data (n=58)**	
Age (years)	69.50 ± 7.80
Male (n, %)	47 (81.0)
Body mass index (kg/m^2^)	29.70 ± 3.57
Body surface area (m^2^)	2.04 ± 0.18
Waist circumference (cm)	104.31 ± 9.00
History of hypertension (n, %)	53 (91.4)
Mean arterial pressure (mmHg)	99.33 ± 13.25
Dyslipidemia n (%)	41 (70.7)
Smoking (n, %)	35 (60.3)
Family history (n, %)	24 (41.4)
Duration diabetes (years)	10.47 ± 8.54
Diabetic polyneuropathy (n, %)	13 (22.4)
Diabetic retinopathy (n, %)	8 (13.8)
Hb_A1C_ (mmol/mol)	52.55 ± 10.26
Fasting glucose (mg/dl)	162.09 ± 53.55
Total cholesterol (mg/dl)	191.64 ± 45.68
LDL-cholesterol (mg/dl)	117.21 ± 36.45
HDL-cholesterol (mg/dl)	43.78 ± 9.76
Triglycerides (mg/dl)	179.57 ± 90.52
GFR (ml/min/1.73 m^2^)	75.26 ± 23.27
NYHA classification (n, %)	
- I	16 (27.6)
- II	28 (48.3)
- III	14 (24.1)
- IV	0 (0)
Medical therapy prior to coronary angiography n (%)	
- Metformin	41 (70.7)
- Insulin	21 (36.2)
- Sulfonylurea	13 (22.4)
- Incretin-based therapy	13 (22.4)
- Statin	41 (70.7)
- ACE-inhibitor/ARB	43 (74.1)
- ß-blocker	45 (77.6)
- ASA	54 (93.1)

The data are presented as mean ± SD or n (%).

Abbreviations: *LDL* low density lipoprotein, *HDL* high density lipoprotein, *GFR* glomerular filtration rate, *ACE* angiotensin - converting enzyme, *ARB* angiotensin receptor blocker, *ASA* acetylsalicylic-acid.

**Table 2:** CMR, OCT and QCA characteristics

	
**CMR data (n = 58)**	
LV-ejection fraction (%)	53.91 ± 8.11
LV-stroke volume (ml)	82.09 ± 20.20
Septum (mm)	12.05 ± 2.00
Lateral wall (mm)	10.09 ± 1.51
LV-ESV (ml)	75.33 ± 35.66
LV-ESV/BSA (ml/m^2^)	36.93 ± 17.12
LV-EDV (ml)	157.62 ± 50.95
LV-ESV/BSA (ml/m^2^)	77.18 ± 23.33
LV-EDD (mm)	51.40 ± 6.19
Left atrium (cm^2^)	23.65 ± 5.43
Right atrium (cm^2^)	21.81 ± 6.35
**OCT data (n = 81)**	
OCT data measurement	
Percent area stenosis (%)	74.37 ± 10.25
Stenosis length (mm)	7.11 ± 5.19
Mean reference area (mm^2^)	6.39 ± 2.11
Minimal luminal diameter (mm)	1.20 ± 0.30
Eccentricity lumen index (%)	23.54 ± 10.84
Minimal luminal area (mm^2^)	1.59 ± 0.80
OCT data plaque characteristic	
Lipid plaque (n, %)	45 (55.5)
- Lipid arc ( ° )	133.24 ± 40.26
- Lipid plaque length (mm)	4.12 ± 2.27
- Minimum fibrous cap thickness (μm)	82.14 ± 24.18
Mean fibrous cap thickness (μm)	132.66 ± 28.91
Calcium plaque (n, %)	58 (71.6)
Fibrous plaque (n, %)	66 (81.5)
Plaque rupture (n, %)	10 (12.3)
Thrombus (n, %)	5 (6.2)
**Angiographic data (QCA) (n = 81)**	
Vessel investigated (n, %)	
- LAD	34 (42.0)
- LCX	20 (24.7)
- RCA	27 (33.3)
Diameter stenosis (%)	61.79 ± 15.64
Lesion length (mm)	8.68 ± 5.10
MLD (mm)	0.93 ± 0.43
RD (mm)	2.45 ± 0.62

The data are presented as mean ± SD or n (%).

Abbreviations: *LAD* left anterior descending coronary artery, *LCX* left circumflex coronary artery, *RCA* right coronary artery, *MLD* minimal luminal diameter, *RD* reference diameter, *LV* left ventricle, *ESV* end systolic volume, *EDD* end diastolic diameter, *EDV* end diastolic volume, *NYHA* New York Heart Association, *BSA* body surface area.

### Association between OCT-derived FCT and CMR-parameters

To investigate the relationship between the FCT of coronary artery lesions and CMR-derived parameters in patients with type 2 diabetes, linear and non-linear regression analysis has been performed between minimal as well as mean FCT on the one hand and CMR-derived LV-dimensions on the other hand.

**Figure 2:**
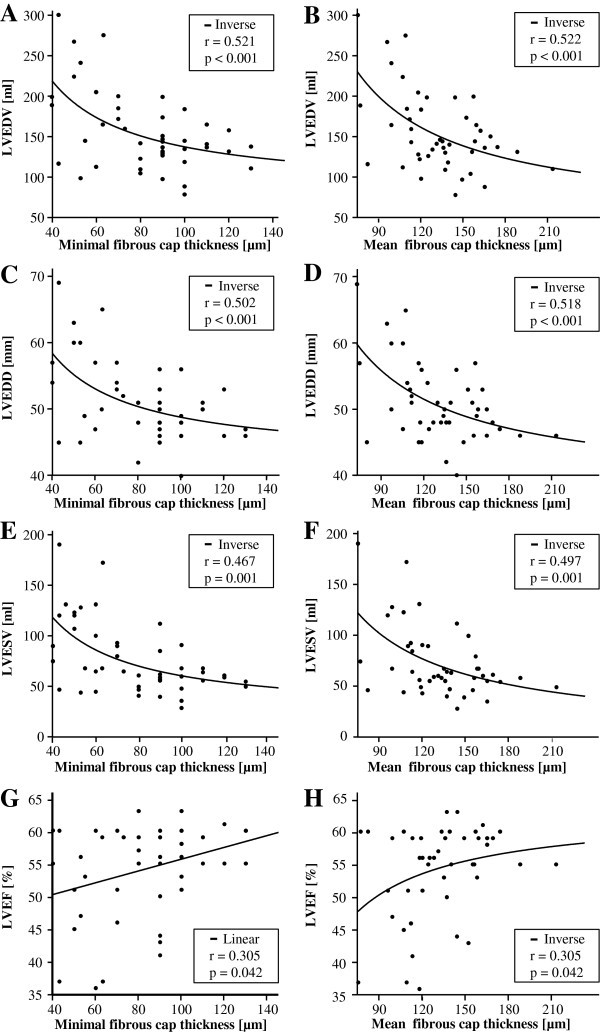
**Association between minimal (A, C, E, G) and mean (B, D, F, H) FCT and CMR-derived LVEDV (A, B), LVEDD (C, D), LVESV (E, F) and LVEF (G, H).** Linear and non-linear regression analysis is displayed with p- and r-values.

There was a significant association between minimal FCT and CMR-derived LVEDV (r = 0.521, p < 0.001), LVESV (r = 0.467, p = 0.001) and LVEDD (r = 0.502, p < 0.001, Figure 2A, C, E).

Similar results were obtained when mean FCT (Figure 2 B, D, F) was related to LVEDV (r = 0.522, p < 0.001), LVESV (r = 0.497, p = 0.001) and LVEDD (r = 0.518, p < 0.001). Furthermore, there was a significant association between both minimal and mean FCT and LVEF (Figure 2 G, F).

### Clinical and OCT-derived plaque characteristics in patients with dilated vs. non-dilated LV

After we found a relationship between minimal FCT and CMR-derived LV-geometry, we compared several clinical, laboratory and morphological plaque parameters between patients with dilated vs. non-dilated LV.

As demonstrated in Table 3, more patients with dilated LV had a history of previous MI (47.1% vs. 14.6%, p = 0.009) and smoking (82.4% vs. 51.2%, p = 0.027) compared to those with non-dilated LV. Furthermore, HDL-cholesterol levels (40.35 ± 5.57 mg/dl vs. 45.20 ± 10.79 mg/dl) were significantly lower in patients with dilated vs. non-dilated LV.

**Table 3:** Patient characteristics between non-dilated and dilated left ventricle

**Clinical data**	**Non-dilated LV**	**Dilated LV**	**p-value**
	**n = 41**	**n = 17**	
Age (years)	69.98 ± 8.16	68.35 ± 6.95	NS
Male (n, %)	31 (75.6)	16 (94.1)	NS
Body mass index (kg/m^2^)	30.05 ± 3.85	28.85 ± 2.71	NS
Body surface area (m²)	2.04 ± 0.17	2.04 ± 0.21	NS
Waist circumference (cm)	104.59 ± 9.65	103.65 ± 7.40	NS
History of hypertension (n, %)	38 (92.7)	15 (88.2)	NS
Mean arterial pressure (mmHg)	100.34 ± 13.96	96.90 ± 11.37	NS
Dyslipidemia (n, %)	28 (68.3)	13 (76.5)	NS
Smoking (n, %)	21 (51.2)	14 (82.4)	0.027
Family History (n, %)	18 (43.9)	6 (35.3)	NS
Duration Diabetes (years)	10.11 ± 8.29	11.32 ± 9.31	NS
Diabetic Polyneuropathy (n, %)	7 (17.1)	6 (35.3)	NS
Diabetic Retinopathy (n, %)	5 (12.2)	3 (17.6)	NS
Previous PCI (n, %)	13 (31.7)	8 (47.1)	NS
Previous MI (n, %)	6 (14.6)	8 (47.1)	0.009
Laboratory parameters			
- Hb_A1C_ (mmol/mol)	53.02 ± 8.49	51.27 ± 14.34	NS
- Fasting glucose (mg/dl)	163.41 ± 57.42	158.88 ± 44.30	NS
- Total cholesterol (mg/dl)	190.83 ± 43.81	193.59 ± 51.29	NS
- LDL-cholesterol (mg/dl)	114.78 ± 32.23	123.06 ± 45.67	NS
- HDL-cholesterol (mg/dl)	45.20 ± 10.79	40.35 ± 5.57	0.029
- Triglycerides (mg/dl)	183.49 ± 96.28	170.12 ± 76.70	NS
- GFR (ml/min/1.73 m²)	76.22 ± 23.64	72.93 ± 22.89	NS
Medical therapy prior to coronary angiography n (%)			
- Metformin (n, %)	30 (73.2)	11 (64.7)	NS
- Insulin (n, %)	14 (34.1)	7 (41.2)	NS
- Sulfonylurea (n, %)	11 (26.8)	2 (11.8)	NS
- Incretin-based therapy (n, %)	11 (26.8)	2 (11.8)	NS
- Statin (n, %)	27 (65.9)	14 (82.4)	NS
- ACE-Inh./ARB (n, %)	32 (78.0)	11 (64.7)	NS
- ß-Blocker (n, %)	31 (75.6)	14 (82.4)	NS
- ASA (n, %)	38 (92.7)	16 (94.1)	NS

The data are presented as mean ± SD or n (%).

Abbreviations: *MI* myocardial infarction, *PCI* percutane coronary intervention and as in Table 1; *NS* for p > 0.1.

Differences in OCT-derived parameters in dilated versus non-dilated LV are depicted in Table 4. There was no difference in lipid arc or lipid plaque length. However, lesions in patients with dilated LV tended to have plaque rupture more frequently (22.7% vs. 8.5%, p = 0.083), supporting the relevance of the relationship observed between FCT and LV-dilatation.

**Table 4:** OCT-derived plaque characteristics in non-dilated and dilated left ventricle

**OCT parameters**	**Non-dilated LV**	**Dilated LV**	**p-value**
	**n = 59**	**n = 22**	
Stenosis (%)	73.97 ± 10.62	75.45 ± 9.33	NS
Stenosis length	6.56 ± 4.52	8.58 ± 6.56	NS
Mean reference area (mm²)	6.38 ± 2.09	6.41 ± 2.22	NS
Minimal luminal diameter(mm)	1.22 ± 0.33	1.17 ± 0.21	NS
Eccentricity lumen index (%)	23.08 ± 10.24	24.77 ± 12.50	NS
Minimal luminal area (mm²)	1.62 ± 0.88	1.49 ± 0.54	NS
Lipid plaque (n, %)	31 (52.5)	14 (63.6)	NS
FCT≤80 μm (n, %)	11 (35.5)	10 (71.4)	0.025
Minimum FCT (μm)	88.52 ± 22.75	68.02 ± 21.76	0.008
Mean FCT (μm)	139.98 ± 28.41	116.44 ± 23.54	0.010
Lipid arc ( ° )	129.26 ± 40.53	142.05 ± 39.67	NS
Lipid plaque length (mm)	4.00 ± 2.29	4.40 ± 2.28	NS
Calcium plaque (n, %)	42 (71.2)	12 (54.5)	NS
Fibrous plaque (n, %)	47 (79.7)	16 (72.7)	NS
Plaque rupture (n, %)	5 (8.5)	5 (22.7)	0.083
Thrombus (n, %)	3 (5.1)	2 (9.1)	NS

The data are presented as mean ± SD or n (%).

Abbreviations: *FCT* fibrous cap thickness and as in Table 2; *NS* for p > 0.1.

### Association between minimal FCT and the presence of LV-dilatation

Given that vulnerable plaques may rupture and cause acute cardiovascular events and as we have observed more previous MIs in patients with dilated LV, we investigated whether the association between FCT and LV-dilatation is dependent on a history of previous MIs. Thus, we calculated the odds ratio (OR) for the lesion’s minimal and mean FCT to predict a dilated LV using logistic regression analysis. Both minimal (OR 0.663, p = 0.013) and mean (OR 0.702, p = 0.017) FCT significantly predicted the presence of LV-dilatation (Table 5). However, both minimal and mean FCT predicted LV-dilatation independent of previous MIs (Table 5, adjustment 1). In an additional adjustment we included all clinical parameters which were significantly different between patients with dilated versus non-dilated LV (Table 5, adjustment 2) in order to determine if minimal and mean FCT predict LV-dilatation independent of these risk factors. In this model both minimal and mean FCT predicted LV-dilatation independently (Table 5, adjustment 2).

**Table 5:** Uni- and multivariable binary logistic regression analysis to predict the presence of LV-dilatation

**OCT parameters**	**Odds ratio (95% CI)**	**p-value**
**Univariable analysis: crude**		
Minimal FCT (10 μm)	0.663 (0.481 – 0.916)	0.013
Mean FCT (10 μm)	0.702 (0.525 – 0.939)	0.017
**Multivariable analysis (Adjusted 1: previous MI)**		
Minimal FCT (10 μm)	0.679 (0.487 – 0.945)	0.022
Mean FCT (10 μm)	0.692 (0.513 – 0.935)	0.016
**Multivariable analysis (Adjusted 2: previous MI, HDL cholesterol, smoking)**		
Minimal FCT (10 μm)	0.696 (0.490 – 0.987)	0.042
Mean FCT (10 μm)	0.691 (0.502 – 0.952)	0.024

Abbreviations: As in Tables 3 and 4.

### CMR-derived parameters predict minimal FCT ≤ 80 μm

After we found significant associations between minimal FCT and CMR-derived LVEDV, LVESV and LVEDD, we tested if these parameters predict minimal FCT ≤ 80 μm, which has recently been suggested to be the optimal OCT-derived cut-off for vulnerable plaques in vivo [22].

**Figure 3:**
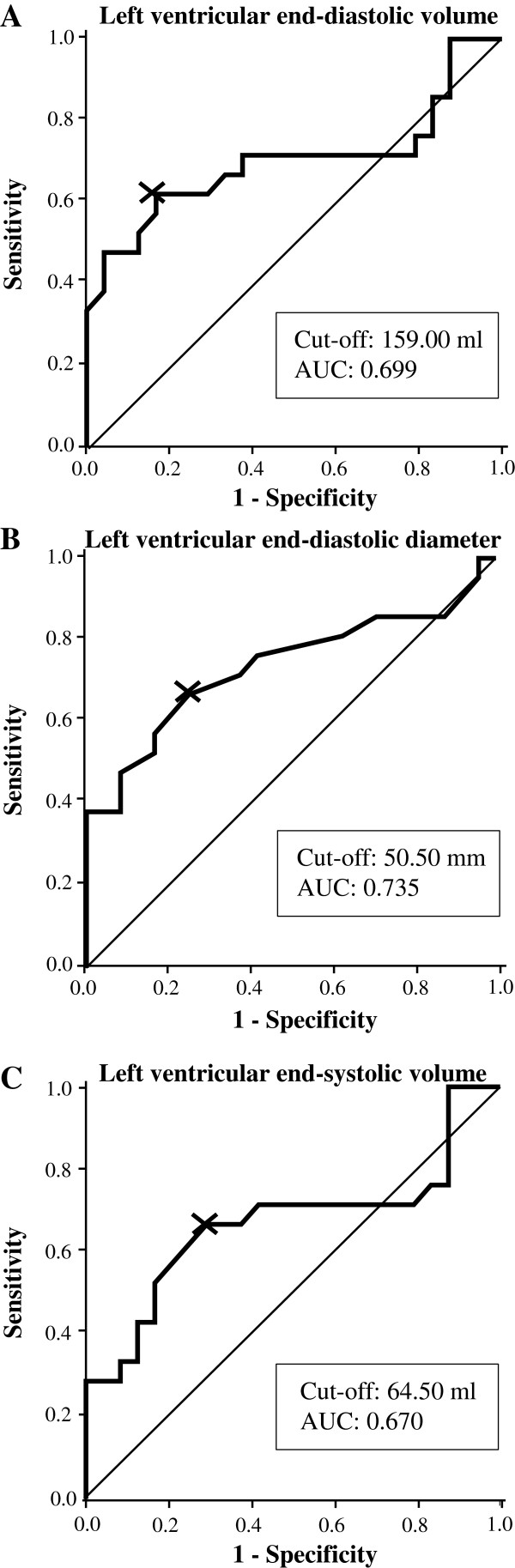
**ROC analysis for CMR-derived A) left ventricular end-diastolic volume B) left ventricular end-diastolic diameter and C) left ventricular end-systolic volume to predict a minimal FCT ≤ 80 μm.** Curves are presented with optimal cut-off values (indicated as X) and the area under the curve (AUC).

Among all CMR-parameters investigated, ROC-analysis revealed that LVEDD was the best predictor for minimal FCT ≤ 80 μm (AUC 0.735, Table 6) with an optimal cut-off value of 50.5 mm. LVEDD, LVEDV and LVESV had a low or moderate overall diagnostic efficiency to predict plaque vulnerability (Figure 3). Specificity at the optimal cut-off values was considerate. These data suggest that patients with dilated LV tend to have more vulnerable plaques whereas both vulnerable and stable plaques are present in patients with non-dilated LV.

**Table 6:** CMR derived left ventricular parameters predict minimal fibrous cap thickness ≤ 80 μm

**Parameter**	**Cut-off**	**Sens**	**Spec**	**PPV**	**NPV**	**AUC**	**95% CI**
LVEDV	159.0 ml	61.9	83.3	76.5	71.4	0.699	0.532-0.867
LVESV	64.5 ml	66.7	70.8	66.7	70.8	0.670	0.500-0.839
LVEDD	50.5 mm	66.7	75.0	70.0	72.0	0.735	0.580-0.891

Abbreviations: *Sens* sensitivity, *Spec* specificity, *PPV* positive predictive value, *NPP* negative predictive value, *AUC* area under the curve, *CI* confidence interval and as in Table 2.

## Discussion

The main findings of this study in patients with type 2 diabetes and stable coronary artery disease are:

1) A decrease of both minimal and mean FCT is associated with increased LV-dilatation as characterized by CMR-derived LVEDV, LVESV and LVEDD.

2) Both minimal and mean FCT are associated with LV-dilatation independent of previous MIs.

3) LVEDD, LVEDV and LVESV predict plaque vulnerability with overall low or moderate diagnostic efficiency but considerate specificity at optimal cut-off values.

A minimal FCT ≤ 80 μm is an approved in vivo predictor of plaque rupture with subsequent acute coronary events [22]. Patients with type 2 diabetes are prone to both, a high prevalence of vulnerable plaques as well as LV-dilatation [1-6]. However, to date nothing is known about the relationship between minimal and mean FCT on the one hand and LV-geometry on the other hand, neither in patients with type 2 diabetes nor in the general population. We extend the current knowledge by demonstrating an association between both minimal and mean FCT with CMR-derived LVEDV, LVESV and LVEDD as markers of LV-dilatation. In addition, we could demonstrate that a large variety of FCT values was present in patients with non-dilated LV, whereas in patients with dilated LV lesions were characterized by a smaller FCT. Thus, CMR-derived parameters at optimal cut-off values displayed low sensitivity in predicting FCT ≤ 80 μm but considerate specificity. Taken together, the data suggest that type 2 diabetic patients with dilated LV are at a particular high risk for vulnerable plaques whereas both vulnerable and stable plaques are present in patients with non-dilated LV.

Plaque rupture causes acute coronary events which subsequently result in ischemic cardiomyopathy with LV-dilatation. Thus, the relationship between FCT and LV-dilatation in patients with type 2 diabetes observed in this study may reflect the relationship between plaque vulnerability and previous MIs. However, although several cardiovascular risk factors including previous MI and HDL-cholesterol were different between patients with dilated versus non-dilated LV, multivariable regression analysis demonstrated that both minimal and mean FCT predict LV-dilatation independent of previous MIs. Moreover, minimal and mean FCT predicted LV-dilatation even when adjusted for further cardiovascular risk factors such as smoking and HDL-cholesterol which were different between patients with dilated versus non-dilated LV. As a direct causal relationship between both minimal and mean FCT on the one hand and LV-dilatation on the other hand seems unlikely, it is tempting to speculate that FCT and LV-dilatation may both be caused by a third pathophysiological entity. This may include microruptures and disturbances in microcirculation. Further candidates which are known to influence both LV-geometry and atherosclerosis include alterations in metabolism, adipokines and cytokines [24-28]. Future studies are needed to determine the underlying mechanisms linking plaque vulnerability and LV-dilatation.

To the best of our knowledge we are first to describe an association between plaque vulnerability as determined by OCT and left ventricular dilatation in patients with type 2 diabetes. However, the correlation between LV geometric parameters and atherosclerosis and the incidence of acute cardiovascular events is not a new concept. Specifically, Chahal et al. have demonstrated in 2279 patients without clinical cardiovascular disease that subclinical carotid plaque disease was related to LV systolic function and LV filling pressure, whereas no significant differences in LV systolic and diastolic volumes were observed between patients with compared to those without carotid disease [29]. Madaj and colleagues have demonstrated an association between LV-mass and coronary artery calcification as determined by computed tomography [30]. Furthermore, the Multiethnic Study of Atherosclerosis (MESA) has found several traditional risk factors of cardiovascular disease to be associated with LVEDV as determined by CMR [31]. Our findings are in line with this study by demonstrating more classical cardiovascular risk factors including smoking, previous MI as well as a decreased HDL-cholesterol in diabetic patients with vs. without LV-dilatation.

Whereas stress-CMR reveals ischemia due to functionally relevant coronary stenosis [32], its diagnostic value in hemodynamically insignificant lesions remains insufficient. However, cardiac events frequently occur in angiographically insignificant stenoses [33] and usually arise from vulnerable lesions with a low FCT. In the light of our data it is tempting to speculate that CMR at rest with the assessment of LV-dilatation may add insights into plaque morphology and vulnerability in high risk patients with type 2 diabetes. Specifically, diagnostic efficiency and sensitivity of CMR-derived LVEDD, LVEDV and LVESV to predict plaque vulnerability was low to moderate with considerate specificity at optimal cut-off values. Thus, CMR-derived parameters of LV-dilatation may be valuable in identifying patients at risk for the presence of vulnerable plaques.

Echocardiography has the advantage of being widely available as well as time- and cost-effective. Moreover, it is the most commonly used imaging technique for the assessment of LV-dilatation. However, CMR for the assessment of LV-dilatation may be superior to echocardiography due to its higher resolution and exacter assessments particularly in overweight and obese patients with type 2 diabetes which is reflected in our own study population with a mean BMI of 29.70 kg/m^2^. It needs to be determined in future studies if our findings can be confirmed when echocardiography instead of CMR is used to assess LV-dilatation.

### Limitations

The present investigation is limited by a small sample size and in 15 patients the determination of a FCT was not possible due to the absence of a lipid-rich plaque. Therefore, for confirmation of our data a large-scale study is required. The sample size may be a limitation particularly for the presented multivariable regression analysis.

Second, patients with reduced kidney function were not included in the study due to ethical issues regarding additional contrast medium necessary for the OCT-investigation. However, a high percentage of those patients with reduced LV-function also have reduced kidney function and our findings may be biased by this fact.Third, although the relationship between LV-geometry and plaque vulnerability is particularly important in cardiovascular high-risk patients with type 2 diabetes, future studies are necessary to determine if the findings of this study can also be translated to patients without type 2 diabetes.

Furthermore, our findings are limited by the fact that in this study OCT was only used in the target vessel due to ethical issues in order to minimize complications associated with the use of OCT.

Moreover, we used the minimal FCT-threshold of 80 μm for defining plaque vulnerability. This value is above the currently accepted consensus of 65 μm that was based on post-mortem studies, where tissue-shrinkage may be a relevant issue [13]. Therefore, we referred to a currently published OCT-investigation which demonstrated that 95% of ruptured lipid-rich plaques presented with a minimal FCT ≤ 80 μm in vivo [22].

## Conclusion

We conclude that in patients with type 2 diabetes and stable coronary artery disease, minimal and mean FCT are associated with LV-dilatation independent of previous MIs. Furthermore, LV-dilatation as determined by CMR may be a useful adjunct to identify patients at risk for vulnerable coronary artery lesions. Future studies are warranted to investigate potential mechanisms linking plaque vulnerability and LV-dilatation in patients with type 2 diabetes.

## Abbreviations

LV: Left ventricle; MI: Myocardial infarction; OCT: Optical coherence tomography; CMR: Cardiac magnetic resonance imaging; FCT: Fibrous cap thickness; LVEDV: Left ventricular-end diastolic volume; IVUS: Intravascular ultrasound; LVEF: Left ventricular ejection fraction; LVEDD: Left ventricular-end diastolic diameter; LVESV: Left ventricular-end systolic volume; OR: Odds ratio; ROC: Receiver-operated curve; AUC: Area under the curve.

## Competing interest

The authors declare that there are no competing interests.

## Authors’ contributions

All authors were involved in reporting the results of this study and all approved the final version of the submitted manuscript. SR, NM and MB contributed in the conception, design and planning of the study. SR, MF, SB, AL, AJ, MH, WL, NM and MB conducted the study and were involved in the collection, analysis and interpretation of the data. MH, WL, SR and MB did the statistical analysis. Manuscript writing: SR and MB. SR is responsible for the overall content and serves as guarantor. All authors read and approved the final manuscript.
